# CPVT: Arrhythmogenesis, Therapeutic Management, and Future Perspectives. A Brief Review of the Literature

**DOI:** 10.3389/fcvm.2019.00092

**Published:** 2019-07-12

**Authors:** Giannis G. Baltogiannis, Dimitrios N. Lysitsas, Giacomo di Giovanni, Giuseppe Ciconte, Juan Sieira, Giulio Conte, Theofilos M. Kolettis, Gian-Battista Chierchia, Carlo de Asmundis, Pedro Brugada

**Affiliations:** ^1^Heart Rhythm Management Centre, Vrije University, Brussels, Belgium; ^2^St. Luke's Hospital Thessaloniki, Thessaloniki, Greece; ^3^Cardiology Department, University of Ioannina, Ioannina, Greece

**Keywords:** channelopathies, CPVT, arrhythmias, genes, sudden death, risk stratification

## Abstract

Catecholaminergic Polymorphic Ventricular Tachycardia (CPVT) is a primary electrical disease characterized by a normal resting electrocardiogram and induction of malignant arrhythmias during adrenergic stress leading to syncope or sudden cardiac death (SCD). CPVT is caused by mutations in the cardiac ryanodine receptor (RyR2) or in the sarcoplasmic reticulum protein calsequestrin 2 genes (*CASQ2*). The RyR2 mutations are responsible for the autosomal dominant form of CPVT, while *CASQ2* mutations are rare and account for the recessive form. These mutations cause a substantial inballance in the homeostasis of intracellular calcium resulting in polymorphic ventricular tachycardia through triggered activity. Beta blockers were for years the cornerstone of therapy in these patients. Sodium channel blockers, especially flecainide, have an additive role in those not responding in beta blockade. Implantation of defibrillators needs a meticulous evaluation since inappropriate shocks may lead to electrical storm. Finally, cardiac sympathetic denervation might also be an alternative therapeutic option. Early identification and risk stratification is of major importance in patients with CPVT. The aim of the present review is to present the arrhythmogenic mechanisms of the disease, the current therapies applied and potential future perspectives.

## Introduction

Catecholaminergic Polymorphic Ventricular Tachycardia (CPVT) is a primary electrical disease characterized by a normal resting electrocardiogram and induction of malignant arrhythmias during adrenergic stress leading to syncope or sudden cardiac death (SCD) (1). CPVT phenotype is a result of mutations of Ryanodine receptor (RyR2) and calsequetrin 2. The RyR2 mutations account for the commonest phenotype and they are inherited through an autosomal dominant manner. CASQ2 mutations represent the recessive form (2, 3).

CPVT is responsible for SCD especially among children and young adults. According to previous reports, the incidence of arrhythmias in CPVT patients was 32% over 8 years (2), but the true frequency of the disease is unknown. This is due to the fact that unlike other inherited channelopathies such as long QT syndrome, it is present not only with a structurally normal heart, but also without resting ECG abnormalities (2).

Beta blockade is the main therapeutic option. Sodium channel blockers, such as flecainide, have an additive role to those not responding to beta blockers, along with left cardiac stellate sympathectomy. Implantable cardiac defribrillators (ICDs) are life -saving therapy for the majority of patients with cardiac channelopathies, however CPVT patients need to be carefully selected, since inappropriate shocks may lead to adrenergic stimulation and electrical storm, despite optimal programming.

## CPVT and Arrhythmogenesis

Arrhythmogenesis in CPVT patients is attributed to mutations in different proteins resulting in bidirectional ventricular tachycardia through different arrhythmogenic mechanisms. Arrhythmias produced by gain-of-function mutations in RyR2 are postulated to result from destabilization of the channel with increased diastolic SR Ca^2+^ leak in ventricular myocytes, leading to delayed afterdepolarizations and triggered activity via the Na+/Ca^2+^ exchanger current. Yet, new evidence has shown that the cardiac Purkinje network appears to be involved in the initiation of bidirectional VT and polymorphic ventricular tachycardia in this disease (4). It is estimated that over 160 mutations causes CPVT 1. Most of them cause a gain of function of the RyR2 channels (1–3), whereas others, such as CASC2 gene, regulate RYR receptor through other proteins (junctin and triadin) resulting to a leakage of Ca in diastole.

According to previous published data (5), gene mutations responsible for CPVT lead to ventricular arrhythmia through the alteration of the Ca2+ homeostasis. Specifically, mutations in the RyR2 and CASQ2 genes lead to a leakage of Ca2+ from the SR in diastole, particularly under adrenergic stress (exercise, emotional stress), resulting in delayed after-depolarizations and therefore vulnerable to ventricular arrhythmias. Other less prevalent gene mutations like KCNJ2, triadin (TRDN), junctin (JCN), calmodulin (CALM1 and CALM2), and NKYRIN-B (6) may predispose to CPVT as well as in the future, other not yet identified genes might be found responsible for the disease.

## Clinical Presentation-Diagnostic Evaluation

Nevertheless, irrespective of the responsible mutation, CPVT is characterized by polymorphic ventricular tachycardia under adrenergic stress. Apart from syncope less specific signs and symptoms, such as dizziness or palpitations might be exerted (7). The first manifestation of the disease occurs during childhood and the majority of patients have experienced syncope episode or cardiac arrest by their adulthood (7). The study of Hayashi et al. (2) depicted that the earliest a CPVT is diagnosed the worse the prognosis is. This can be attributed, at least in part, to the fact that children performing strenuous physical activities are more sensitive to external stimulations (children have more opportunities to engage in strenuous activities), (1) patients with more severe forms of CPVT will be diagnosed earlier, and (2) beta-blockers are frequently underdosed in children if based on weight given increased hepatic clearance. Sudden cardiac death or syncope in first degree family members is detected in one third of CPVT patients (8). Despite its life threatening nature, CPVT remains often unnoticed. This is due to normal baseline electrocardiograms on top of incomplete penetrance (8, 9) and thus variable expressivity. Some authors have reported bradycardia, and others have observed U waves in electrocardiograms (10). CPVT is unmasked by a treadmill stress test (11). When patients start exercising ventricular ectopy develops, increasing in complexity as the heart rate increases. Specifically, dynamic exercise during a BRUCE protocol induces premature ventricular complexes that may degenerate to more complex ventricular tachyarrhythmias or even sustained VT (12, 13).

## Therapeutic Management

### Beta Blockers

Therapeutic management for patients with CPVT includes beta blockers without intrinsic sympathomimetic activity. Nadolol is the beta-blocker of choice in a high dosage, 1–2 mg/kg. The incidence of arrhythmic events in CPVT patients on beta-blockers is still high. Other non-selective beta-blockers are equally effective especially propranolol. Clinical follow up with holter monitoring and treadmill stress test should be performed so that the optimal therapy is adjusted (14).

In the study of Priori et al. (14), there is significantly lower incidence of SCD in patients on beta-blockers. Hence, the event rates in the patients on therapy were not negligible. This could be attributed to poor therapy compliance. Priori et al. suggest that taking different beta bockers than nadolol could be associated with higher incidence rates. Furthermore, data from treadmill stress tests reveal that it is not the ultimate tool during follow up, despite the fact that it is widely used as a diagnostic tool, due to low sensitivity and specificity (14).

Chatzidou et al. (15) suggested that patients presenting with electrical storm independently of the underlying mechanism should be treated with oral propranolol as the preferred beta-blocker agent.

### Flecainide

The study of van de Weerf et al. (16) supports the use of flecainide on top of beta blockers as it reduces ventricular arrhythmias during exercise. This is of major importance, since several studies have demonstrated a significant event rate despite conventional therapy (2, 9, 17–23). Therefore, adding flecainide in combination with β-blocker therapy should be considered.

In CPVT the rise of intracellular Ca2+ activates the electrogenic Na+/Ca2+ exchanger (NCX), which produces a transient inward current (ITi). ITi generates delayed afterdepolarizations, which can lead to triggered activity, and the initiation of ventricular arrhythmias (24). Flecainide directly targets the molecular defect in CPVT by inhibiting RyR2 channels and preventing arrhythmogenic Ca2+ waves. Flecainide's Na+ channel blockade further reduces the rate of triggered beats (5, 25, 26). This dual action could explain why flecainide is so effective in severe CPVT and provides a rationale for combination therapy with β-blockers. The rationale for flecainide use for treatment of CPVT is supported by *in vitro* studies demonstrating that flecainide blocks RyR2 in lipid bilayers (27) suppresses calcium waves in CASQ2-knockout myocytes, abolishes delayed afterdepolarization–mediated triggered activity, and reduces exercise induced ventricular arrhythmias inCASQ2 and RYR2 mouse models. The efficacy of flecainide in human patients with CPVT has been demonstrated in the 3 retrospective cohorts. Kannankeril et al. (25) supported that a median dosage of 300 mg/d was required to achieve target trough drug levels. One could speculate that chronotropic incompetence from combination therapy with β-blocker plus flecainide would result in lower levels of exertion during exercise and thus a lower arrhythmia score. However, maximal workload achieved during each exercise test did not differ significantly, suggesting similar levels of effort across the three exercise tests.

Liu et al. support that the antiarrhythmic effect of flecainide is that it reduces the availability of sodium channels, thus preventing the development of triggered APs (28).

Radwanski et al. suggested that flecainide may exert its antiarrhythmic action by antagonizing catecholamine-dependent augmentation of Na+ influx via sodium channel isoforms, and Nav1.6 in particular (29).

### Left Cardiac Sympathetic Denervation

In patients who are refractory to maximal pharmacologic treatment, left cardiac sympathetic denervation (LCSD) could be an alternative, with significant reduction in arrhythmic events, as noted by De Ferrari et al. (30). However, the procedure is not widely available and is associated with complications such as pneumothorax and Horner syndrome (30).

### Implantable Cardioverter Defibrillator

An ICD, usually the ultimate solution for primary or secondary prevention of SCD for other channelopathies should be used in CPVT patients who, despite optimal medical management or/and other therapies such as left cardiac sympathectomy continue to be in danger. Patients who have experienced an aborted cardiac arrest before the initiation of therapy, should be on medical therapy together with an ICD implantation (31). Hence, implantation of an ICD is a technical challenge in a pediatric population and problems such as inappropriate shocks, proarrhythmic effects of the ICD, and the need for a lifetime protection requiring multiple reinterventions should be addressed when the decision is taken (32).

Current knowledge suggests an ICD implantation to survivors of cardiac arrest, or when syncope or sustained VT persists despite maximal tolerable beta blockade (14). Nevertheless, ICDs should be used with caution since they can trigger electrical storms via a vicious circle of adrenergic stimulation by the delivered shocks in CPVT patients (31).

## Future Perspectives

Arrhythmic events among probands and family members are still challenging. To the best of our knowledge a reliable risk stratification tool lacks in patients with CPVT. Cardiac events may happen in previously asymptomatic mutations carriers, even with negative treadmill tests. Consequently, there is an emerging need to better clarify the individuals at risk for future events. Apart from meticulous family screening mutations carriers identified should be treated with beta-blockers even after a negative exercise test, which can change with time (14).

All CPVT patients should have a genetic diagnosis, that might further assist to an individualized treatment. Moreover, in concordnace with other cardiac channelopathies a risk stratification model should be developed in order to identify patients at higher risk. Novel therapeutic strategies are also needed, especially for non-responders to current therapeutic options. An interesting perspective for the future is gene-therapy, which entails a therapy targeted at correcting the genetic mutation responsible for the disease (33, 34).

## Conclusion

Current evidence suggests that risk stratification in mutation carriers is mandatory along with new therapies, especially for young patients, who survived aborted cardiac arrest or those with poor beta-blocker efficacy.

Beta-blockers is still the cornerstone in treating CPVT patients. ICDs should be considered only as a last resort taking into account their potentially harmful effect in CPVT patients, especially in children.

## Author Contributions

GB has authored this paper. DL has made substantial changes on the draft. GdG and GCi edited bibliography. JS, GCo, G-BC, and CdA made useful remarks on the first manuscript. TK edited English language. PB edited the paper.

### Conflict of Interest Statement

The authors declare that the research was conducted in the absence of any commercial or financial relationships that could be construed as a potential conflict of interest.
